# Supplementary material for: Hot spot prediction in protein-protein interactions by an ensemble system
Source: BMC Syst Biol. 2018 Dec 31;12(Suppl 9):132. doi: 10.1186/s12918-018-0665-8 (PMC6311905; doi:10.1186/s12918-018-0665-8)
Supplement: Supplementary file 2 — All relevant MATLAB code. A simple Matlab implement of our predictor. (ZIP 19,742 kb) [file 12918_2018_665_MOESM2_ESM.zip › Additional file 2/Read ASA/ASA website/dbMPIKT/4LRXA.html]

 NetSurfP - Protein Surface Accessibility and Secondary Structure Predictions

  

|  |  |  |
| --- | --- | --- |
|  |  | NetSurfP - Protein Surface Accessibility and Secondary Structure Predictions  Technical University of Denmark |

  


---

```
# For publication of results, please cite:
# A generic method for assignment of reliability scores applied to solvent accessibility predictions.
# Bent Petersen, Thomas Nordahl Petersen, Pernille Andersen, Morten Nielsen and Claus Lundegaard
# BMC Structural Biology 2009, 9:51 doi:10.1186/1472-6807-9-51
#
# Column 1: Class assignment - B for buried or E for Exposed - Threshold: 25% exposure, but not based on RSA
# Column 2: Amino acid
# Column 3: Sequence name
# Column 4: Amino acid number
# Column 5: Relative Surface Accessibility - RSA
# Column 6: Absolute Surface Accessibility
# Column 7: Z-fit score for RSA prediction
# Column 8: Probability for Alpha-Helix
# Column 9: Probability for Beta-strand
# Column 10: Probability for Coil
E M  4LRXA                  1    0.594 118.799  -0.764   0.003   0.003   0.994
E K  4LRXA                  2    0.488 100.443   1.037   0.052   0.084   0.864
E K  4LRXA                  3    0.444  91.290   1.145   0.118   0.150   0.732
B L  4LRXA                  4    0.187  34.313  -0.424   0.113   0.087   0.800
B I  4LRXA                  5    0.223  41.237  -0.077   0.053   0.043   0.903
E N  4LRXA                  6    0.380  55.617  -0.511   0.018   0.019   0.964
E D  4LRXA                  7    0.481  69.384   0.873   0.016   0.005   0.979
B V  4LRXA                  8    0.201  30.848  -0.413   0.321   0.003   0.675
E Q  4LRXA                  9    0.613 109.410   0.213   0.321   0.003   0.675
E D  4LRXA                 10    0.430  61.905   1.421   0.622   0.015   0.363
B V  4LRXA                 11    0.065   9.914   1.320   0.858   0.002   0.139
B L  4LRXA                 12    0.026   4.742   1.080   0.970   0.001   0.030
E D  4LRXA                 13    0.405  58.389   1.327   0.970   0.001   0.030
E E  4LRXA                 14    0.331  57.826   1.167   0.988   0.000   0.012
B Q  4LRXA                 15    0.044   7.841   0.862   0.988   0.000   0.012
B L  4LRXA                 16    0.029   5.383   0.479   0.970   0.001   0.030
E A  4LRXA                 17    0.373  41.061   1.382   0.970   0.001   0.030
B G  4LRXA                 18    0.058   4.549  -1.567   0.988   0.000   0.012
B L  4LRXA                 19    0.033   6.024   1.059   0.970   0.001   0.030
B A  4LRXA                 20    0.052   5.763   0.099   0.975   0.003   0.022
E K  4LRXA                 21    0.352  72.509   0.968   0.938   0.007   0.055
B A  4LRXA                 22    0.066   7.295  -0.254   0.802   0.014   0.185
B H  4LRXA                 23    0.151  27.412   1.054   0.246   0.004   0.750
E P  4LRXA                 24    0.647  91.781   0.679   0.053   0.005   0.942
E S  4LRXA                 25    0.620  72.699   0.522   0.018   0.019   0.964
B L  4LRXA                 26    0.099  18.182   1.032   0.005   0.336   0.660
E T  4LRXA                 27    0.357  49.488   0.613   0.003   0.718   0.279
B L  4LRXA                 28    0.301  55.131   0.586   0.018   0.846   0.136
E H  4LRXA                 29    0.300  54.534   0.877   0.021   0.756   0.223
E Q  4LRXA                 30    0.465  83.120  -0.531   0.005   0.262   0.733
E D  4LRXA                 31    0.739 106.504  -0.725   0.005   0.015   0.979
E P  4LRXA                 32    0.483  68.566  -0.371   0.005   0.015   0.979
B V  4LRXA                 33    0.229  35.274   0.322   0.005   0.262   0.733
B Y  4LRXA                 34    0.064  13.570   0.655   0.001   0.900   0.099
B V  4LRXA                 35    0.014   2.167   1.393   0.001   0.959   0.040
B T  4LRXA                 36    0.060   8.294   0.551   0.001   0.959   0.040
B R  4LRXA                 37    0.098  22.419   0.697   0.001   0.900   0.099
E A  4LRXA                 38    0.265  29.148   0.603   0.004   0.420   0.576
E D  4LRXA                 39    0.618  89.068   0.627   0.004   0.138   0.858
E A  4LRXA                 40    0.428  47.177  -0.162   0.005   0.015   0.979
E P  4LRXA                 41    0.554  78.570  -0.516   0.005   0.015   0.979
E V  4LRXA                 42    0.608  93.388  -0.040   0.018   0.019   0.964
E A  4LRXA                 43    0.646  71.222   0.773   0.005   0.015   0.979
E G  4LRXA                 44    0.445  35.021  -0.044   0.005   0.015   0.979
B K  4LRXA                 45    0.209  42.991   1.350   0.005   0.262   0.733
B V  4LRXA                 46    0.034   5.195   0.343   0.002   0.816   0.182
B A  4LRXA                 47    0.039   4.331   0.176   0.001   0.959   0.040
B L  4LRXA                 48    0.028   5.127   0.840   0.001   0.959   0.040
B L  4LRXA                 49    0.026   4.687   1.046   0.001   0.959   0.040
B S  4LRXA                 50    0.033   3.903  -0.228   0.002   0.816   0.182
B G  4LRXA                 51    0.182  14.347  -0.915   0.005   0.336   0.660
B G  4LRXA                 52    0.203  15.976  -1.409   0.018   0.088   0.893
B G  4LRXA                 53    0.166  13.104  -1.793   0.018   0.088   0.893
B S  4LRXA                 54    0.195  22.831  -1.341   0.052   0.084   0.864
B G  4LRXA                 55    0.215  16.944  -1.847   0.053   0.043   0.903
B H  4LRXA                 56    0.175  31.778  -0.449   0.115   0.016   0.868
B E  4LRXA                 57    0.177  30.904   0.297   0.115   0.016   0.868
B P  4LRXA                 58    0.101  14.275  -0.280   0.113   0.043   0.844
B M  4LRXA                 59    0.083  16.568  -0.755   0.113   0.087   0.800
B H  4LRXA                 60    0.079  14.297  -0.300   0.118   0.150   0.732
B C  4LRXA                 61    0.057   8.003  -0.441   0.118   0.150   0.732
B G  4LRXA                 62    0.044   3.494  -2.099   0.125   0.227   0.648
B Y  4LRXA                 63    0.073  15.557   0.102   0.135   0.317   0.548
B I  4LRXA                 64    0.066  12.284  -0.409   0.135   0.317   0.548
B G  4LRXA                 65    0.091   7.162  -0.521   0.118   0.150   0.732
E Q  4LRXA                 66    0.426  76.030   0.763   0.053   0.043   0.903
B G  4LRXA                 67    0.188  14.780  -2.083   0.053   0.043   0.903
B M  4LRXA                 68    0.084  16.808   0.162   0.113   0.087   0.800
B L  4LRXA                 69    0.064  11.663  -0.869   0.056   0.142   0.802
B S  4LRXA                 70    0.230  26.991  -0.612   0.056   0.142   0.802
B G  4LRXA                 71    0.058   4.541  -1.648   0.064   0.216   0.721
B A  4LRXA                 72    0.083   9.103  -0.896   0.021   0.279   0.699
B C  4LRXA                 73    0.071   9.912  -0.575   0.022   0.359   0.619
B P  4LRXA                 74    0.096  13.637  -0.016   0.022   0.359   0.619
B G  4LRXA                 75    0.080   6.265  -1.058   0.019   0.141   0.840
E E  4LRXA                 76    0.259  45.195  -0.534   0.019   0.141   0.840
B I  4LRXA                 77    0.071  13.061  -0.212   0.021   0.279   0.699
B F  4LRXA                 78    0.111  22.177  -0.751   0.005   0.336   0.660
B T  4LRXA                 79    0.122  16.866  -0.895   0.004   0.197   0.799
B S  4LRXA                 80    0.122  14.287  -0.658   0.004   0.085   0.910
B P  4LRXA                 81    0.173  24.520  -0.296   0.005   0.015   0.979
E T  4LRXA                 82    0.255  35.313  -0.171   0.016   0.005   0.979
B P  4LRXA                 83    0.093  13.239  -0.898   0.923   0.002   0.076
E D  4LRXA                 84    0.555  79.990   0.949   0.970   0.001   0.030
B K  4LRXA                 85    0.178  36.656   1.161   0.970   0.001   0.030
B I  4LRXA                 86    0.015   2.830   1.435   0.988   0.000   0.012
B F  4LRXA                 87    0.142  28.479   1.239   0.988   0.000   0.012
E E  4LRXA                 88    0.420  73.304   1.719   0.988   0.000   0.012
B C  4LRXA                 89    0.030   4.212   0.389   0.970   0.001   0.030
B A  4LRXA                 90    0.036   3.945   0.012   0.975   0.003   0.022
E M  4LRXA                 91    0.426  85.343   1.397   0.938   0.007   0.055
E Q  4LRXA                 92    0.441  78.709   0.630   0.802   0.014   0.185
B V  4LRXA                 93    0.048   7.378  -0.204   0.430   0.016   0.555
E D  4LRXA                 94    0.538  77.468   0.049   0.113   0.043   0.844
E G  4LRXA                 95    0.611  48.125  -0.355   0.018   0.019   0.964
E G  4LRXA                 96    0.672  52.918  -1.569   0.016   0.005   0.979
E E  4LRXA                 97    0.407  71.050   0.971   0.005   0.015   0.979
B G  4LRXA                 98    0.065   5.131  -0.894   0.005   0.262   0.733
B V  4LRXA                 99    0.028   4.334   0.900   0.001   0.900   0.099
B L  4LRXA                100    0.022   3.955   0.997   0.001   0.959   0.040
B L  4LRXA                101    0.029   5.292   1.331   0.000   0.983   0.017
B I  4LRXA                102    0.019   3.570   1.397   0.001   0.959   0.040
B I  4LRXA                103    0.028   5.217   0.754   0.001   0.900   0.099
B K  4LRXA                104    0.132  27.132   0.241   0.023   0.655   0.322
B N  4LRXA                105    0.107  15.636   0.093   0.021   0.279   0.699
B Y  4LRXA                106    0.158  33.786   0.167   0.113   0.087   0.800
B T  4LRXA                107    0.224  31.041  -0.991   0.268   0.043   0.689
B G  4LRXA                108    0.247  19.439  -1.215   0.257   0.016   0.727
B D  4LRXA                109    0.149  21.500  -0.695   0.339   0.016   0.645
B I  4LRXA                110    0.169  31.191   0.547   0.455   0.046   0.498
B L  4LRXA                111    0.149  27.355  -0.010   0.455   0.046   0.498
B N  4LRXA                112    0.131  19.164  -0.133   0.561   0.047   0.393
B F  4LRXA                113    0.057  11.480   0.139   0.938   0.007   0.055
E E  4LRXA                114    0.267  46.697   0.123   0.938   0.007   0.055
B T  4LRXA                115    0.198  27.518   0.386   0.923   0.002   0.076
B A  4LRXA                116    0.028   3.130  -0.030   0.970   0.001   0.030
B T  4LRXA                117    0.107  14.827   0.679   0.970   0.001   0.030
E E  4LRXA                118    0.388  67.766   0.970   0.970   0.001   0.030
B L  4LRXA                119    0.203  37.078   1.542   0.970   0.001   0.030
B L  4LRXA                120    0.037   6.775  -0.218   0.970   0.001   0.030
E H  4LRXA                121    0.584 106.284   1.678   0.923   0.002   0.076
E D  4LRXA                122    0.624  89.947  -0.428   0.694   0.003   0.303
E S  4LRXA                123    0.501  58.752   0.459   0.246   0.004   0.750
E G  4LRXA                124    0.758  59.631  -0.595   0.003   0.003   0.994
B V  4LRXA                125    0.133  20.488   1.536   0.005   0.045   0.951
E K  4LRXA                126    0.554 114.020   1.885   0.004   0.420   0.576
B V  4LRXA                127    0.043   6.609   0.980   0.002   0.816   0.182
E T  4LRXA                128    0.310  42.969   1.067   0.001   0.900   0.099
B T  4LRXA                129    0.142  19.626   1.381   0.001   0.959   0.040
B V  4LRXA                130    0.020   3.089   1.559   0.001   0.959   0.040
B V  4LRXA                131    0.068  10.405   0.922   0.001   0.959   0.040
B I  4LRXA                132    0.028   5.143   0.347   0.002   0.816   0.182
B D  4LRXA                133    0.164  23.704   0.401   0.021   0.279   0.699
B D  4LRXA                134    0.163  23.546  -0.161   0.056   0.142   0.802
B D  4LRXA                135    0.281  40.420  -0.647   0.056   0.142   0.802
B V  4LRXA                136    0.079  12.127  -0.035   0.064   0.216   0.721
B A  4LRXA                137    0.181  19.968  -1.358   0.056   0.142   0.802
B V  4LRXA                138    0.215  32.984  -0.599   0.052   0.084   0.864
E K  4LRXA                139    0.477  98.201   0.498   0.053   0.043   0.903
E D  4LRXA                140    0.551  79.356   0.524   0.053   0.043   0.903
E S  4LRXA                141    0.601  70.390  -0.731   0.053   0.043   0.903
E L  4LRXA                142    0.736 134.853  -0.670   0.058   0.017   0.925
E Y  4LRXA                143    0.531 113.453  -0.318   0.053   0.043   0.903
E T  4LRXA                144    0.516  71.611  -0.090   0.053   0.043   0.903
E A  4LRXA                145    0.502  55.331  -0.797   0.053   0.043   0.903
E G  4LRXA                146    0.427  33.573  -0.418   0.053   0.043   0.903
B R  4LRXA                147    0.179  40.991  -0.214   0.113   0.043   0.844
B R  4LRXA                148    0.164  37.648  -0.260   0.113   0.087   0.800
B G  4LRXA                149    0.079   6.194  -1.171   0.268   0.043   0.689
B V  4LRXA                150    0.043   6.655   0.226   0.455   0.046   0.498
B A  4LRXA                151    0.024   2.612   0.211   0.622   0.015   0.363
B N  4LRXA                152    0.034   4.919  -0.788   0.802   0.014   0.185
B T  4LRXA                153    0.058   8.003   0.397   0.938   0.007   0.055
B V  4LRXA                154    0.031   4.719   0.870   0.975   0.003   0.022
B L  4LRXA                155    0.046   8.423   1.148   0.975   0.003   0.022
B I  4LRXA                156    0.022   4.089   1.146   0.970   0.001   0.030
B E  4LRXA                157    0.064  11.093   0.940   0.970   0.001   0.030
B K  4LRXA                158    0.107  21.907   1.087   0.970   0.001   0.030
B L  4LRXA                159    0.031   5.694   0.580   0.988   0.000   0.012
B V  4LRXA                160    0.031   4.719   0.458   0.970   0.001   0.030
B G  4LRXA                161    0.058   4.572  -0.458   0.970   0.001   0.030
B A  4LRXA                162    0.067   7.350   0.265   0.970   0.001   0.030
B A  4LRXA                163    0.082   8.981   0.440   0.970   0.001   0.030
B A  4LRXA                164    0.072   7.901   0.202   0.923   0.002   0.076
E E  4LRXA                165    0.524  91.595   0.379   0.782   0.003   0.216
E R  4LRXA                166    0.598 136.873   1.437   0.406   0.004   0.590
E G  4LRXA                167    0.686  53.996  -1.156   0.053   0.005   0.942
B D  4LRXA                168    0.252  36.256   1.346   0.016   0.005   0.979
E S  4LRXA                169    0.481  56.397   1.321   0.018   0.019   0.964
B L  4LRXA                170    0.097  17.669   1.024   0.923   0.002   0.076
E D  4LRXA                171    0.629  90.668   1.500   0.970   0.001   0.030
E A  4LRXA                172    0.423  46.582   2.269   0.970   0.001   0.030
B C  4LRXA                173    0.017   2.415   1.610   0.970   0.001   0.030
B A  4LRXA                174    0.191  21.092   1.839   0.970   0.001   0.030
E E  4LRXA                175    0.540  94.408   1.996   0.970   0.001   0.030
B L  4LRXA                176    0.073  13.348   1.179   0.988   0.000   0.012
B G  4LRXA                177    0.019   1.495   0.903   0.988   0.000   0.012
E R  4LRXA                178    0.440 100.783   1.911   0.988   0.000   0.012
E K  4LRXA                179    0.363  74.731   1.982   0.970   0.001   0.030
B L  4LRXA                180    0.021   3.900   1.010   0.938   0.007   0.055
B N  4LRXA                181    0.091  13.308   0.297   0.879   0.010   0.111
E N  4LRXA                182    0.600  87.781   1.075   0.694   0.003   0.303
E Q  4LRXA                183    0.319  56.938  -0.014   0.430   0.016   0.555
B G  4LRXA                184    0.049   3.880  -0.083   0.216   0.235   0.548
B H  4LRXA                185    0.163  29.595   0.264   0.087   0.683   0.230
B S  4LRXA                186    0.068   7.993  -1.062   0.018   0.846   0.136
B I  4LRXA                187    0.037   6.808   0.426   0.018   0.846   0.136
B G  4LRXA                188    0.034   2.660  -1.211   0.018   0.846   0.136
B I  4LRXA                189    0.043   7.992  -0.012   0.021   0.756   0.223
B A  4LRXA                190    0.041   4.474  -2.263   0.023   0.655   0.322
B L  4LRXA                191    0.101  18.530  -0.513   0.022   0.359   0.619
B G  4LRXA                192    0.240  18.880  -0.255   0.020   0.205   0.775
B A  4LRXA                193    0.269  29.589  -1.368   0.019   0.141   0.840
B C  4LRXA                194    0.089  12.496  -0.627   0.020   0.205   0.775
B T  4LRXA                195    0.226  31.360  -0.288   0.021   0.279   0.699
B V  4LRXA                196    0.115  17.614   0.504   0.020   0.205   0.775
B P  4LRXA                197    0.247  35.049  -0.268   0.019   0.141   0.840
E A  4LRXA                198    0.367  40.399  -1.593   0.018   0.047   0.935
E A  4LRXA                199    0.420  46.262  -0.222   0.018   0.088   0.893
E G  4LRXA                200    0.587  46.173  -1.554   0.018   0.047   0.935
E K  4LRXA                201    0.497 102.212   0.136   0.018   0.047   0.935
E P  4LRXA                202    0.516  73.277  -0.720   0.018   0.047   0.935
E S  4LRXA                203    0.516  60.487  -1.167   0.018   0.088   0.893
B F  4LRXA                204    0.161  32.252  -0.425   0.020   0.205   0.775
E T  4LRXA                205    0.584  80.931   1.334   0.021   0.279   0.699
B L  4LRXA                206    0.092  16.845   0.549   0.019   0.141   0.840
E A  4LRXA                207    0.505  55.640   0.730   0.018   0.047   0.935
E D  4LRXA                208    0.700 100.928  -0.197   0.058   0.017   0.925
E N  4LRXA                209    0.475  69.540  -0.387   0.053   0.043   0.903
E E  4LRXA                210    0.260  45.352   1.048   0.064   0.216   0.721
B M  4LRXA                211    0.073  14.527   0.199   0.023   0.655   0.322
B E  4LRXA                212    0.119  20.842  -0.155   0.021   0.756   0.223
B F  4LRXA                213    0.085  17.100   0.232   0.087   0.683   0.230
B G  4LRXA                214    0.052   4.061  -1.560   0.022   0.552   0.426
B V  4LRXA                215    0.084  12.957  -0.103   0.022   0.359   0.619
B G  4LRXA                216    0.073   5.769  -1.392   0.022   0.359   0.619
B I  4LRXA                217    0.064  11.914   0.028   0.021   0.279   0.699
B H  4LRXA                218    0.134  24.284  -0.068   0.019   0.141   0.840
B G  4LRXA                219    0.253  19.895  -0.922   0.018   0.047   0.935
B E  4LRXA                220    0.183  31.988  -0.006   0.018   0.019   0.964
E P  4LRXA                221    0.444  62.975  -0.011   0.018   0.019   0.964
B G  4LRXA                222    0.192  15.103  -1.360   0.018   0.047   0.935
B I  4LRXA                223    0.216  40.052   0.301   0.019   0.141   0.840
E D  4LRXA                224    0.534  77.007  -0.057   0.066   0.296   0.638
E R  4LRXA                225    0.460 105.271   0.608   0.021   0.279   0.699
E R  4LRXA                226    0.323  73.944   0.470   0.022   0.359   0.619
E P  4LRXA                227    0.626  88.872   0.454   0.021   0.279   0.699
B F  4LRXA                228    0.200  40.120   0.500   0.004   0.085   0.910
E S  4LRXA                229    0.525  61.530  -0.197   0.005   0.015   0.979
E S  4LRXA                230    0.437  51.252   0.150   0.005   0.015   0.979
B L  4LRXA                231    0.048   8.844  -1.114   0.694   0.003   0.303
E D  4LRXA                232    0.627  90.423   0.902   0.923   0.002   0.076
E Q  4LRXA                233    0.501  89.496   1.675   0.970   0.001   0.030
B T  4LRXA                234    0.045   6.269   0.115   0.970   0.001   0.030
B V  4LRXA                235    0.023   3.535   1.026   0.970   0.001   0.030
E D  4LRXA                236    0.438  63.058   2.177   0.988   0.000   0.012
E E  4LRXA                237    0.350  61.145   2.141   0.970   0.001   0.030
B M  4LRXA                238    0.029   5.843   0.560   0.970   0.001   0.030
B F  4LRXA                239    0.043   8.610   0.310   0.970   0.001   0.030
E D  4LRXA                240    0.540  77.872   1.641   0.970   0.001   0.030
E T  4LRXA                241    0.356  49.363   1.711   0.923   0.002   0.076
B L  4LRXA                242    0.029   5.237   0.159   0.858   0.002   0.139
B L  4LRXA                243    0.133  24.352   0.337   0.600   0.003   0.397
E V  4LRXA                244    0.647  99.413  -0.223   0.406   0.004   0.590
E N  4LRXA                245    0.594  86.947  -1.063   0.246   0.004   0.750
E G  4LRXA                246    0.374  29.442  -1.148   0.176   0.004   0.820
E S  4LRXA                247    0.519  60.780  -1.127   0.181   0.016   0.803
E Y  4LRXA                248    0.407  86.933  -0.807   0.257   0.016   0.727
E H  4LRXA                249    0.490  89.076  -1.392   0.268   0.043   0.689
E R  4LRXA                250    0.295  67.486  -0.993   0.268   0.043   0.689
E T  4LRXA                251    0.430  59.586  -1.508   0.278   0.093   0.628
B L  4LRXA                252    0.249  45.610  -1.462   0.307   0.165   0.527
E R  4LRXA                253    0.392  89.791  -1.131   0.307   0.165   0.527
E F  4LRXA                254    0.389  78.032  -1.501   0.278   0.093   0.628
B W  4LRXA                255    0.256  61.664  -1.480   0.278   0.093   0.628
E D  4LRXA                256    0.359  51.790  -1.702   0.354   0.048   0.598
E Y  4LRXA                257    0.425  90.780  -1.381   0.455   0.046   0.498
E Q  4LRXA                258    0.410  73.155  -1.601   0.455   0.046   0.498
E Q  4LRXA                259    0.358  64.010  -1.463   0.354   0.048   0.598
E G  4LRXA                260    0.394  31.031  -1.624   0.354   0.048   0.598
E S  4LRXA                261    0.398  46.634  -1.260   0.354   0.048   0.598
E W  4LRXA                262    0.318  76.407  -1.435   0.386   0.097   0.517
E Q  4LRXA                263    0.452  80.817  -1.117   0.386   0.097   0.517
E E  4LRXA                264    0.421  73.514  -1.269   0.386   0.097   0.517
E E  4LRXA                265    0.442  77.200  -1.388   0.354   0.048   0.598
E Q  4LRXA                266    0.444  79.298  -1.684   0.354   0.048   0.598
E Q  4LRXA                267    0.469  83.746  -1.072   0.386   0.097   0.517
E T  4LRXA                268    0.400  55.522  -1.518   0.278   0.093   0.628
E K  4LRXA                269    0.448  92.071  -1.055   0.191   0.086   0.723
E Q  4LRXA                270    0.316  56.420  -0.577   0.113   0.087   0.800
E P  4LRXA                271    0.545  77.307  -0.382   0.053   0.043   0.903
B L  4LRXA                272    0.117  21.514  -0.538   0.053   0.043   0.903
E Q  4LRXA                273    0.579 103.338  -0.587   0.018   0.019   0.964
E S  4LRXA                274    0.605  70.871  -0.598   0.018   0.019   0.964
E G  4LRXA                275    0.543  42.750  -1.352   0.016   0.005   0.979
B D  4LRXA                276    0.217  31.313  -0.018   0.018   0.047   0.935
E R  4LRXA                277    0.384  87.867   1.566   0.004   0.616   0.381
B V  4LRXA                278    0.030   4.580   0.857   0.001   0.959   0.040
B I  4LRXA                279    0.017   3.127   1.495   0.001   0.959   0.040
B A  4LRXA                280    0.017   1.851   1.478   0.000   0.983   0.017
B L  4LRXA                281    0.030   5.530   0.773   0.001   0.959   0.040
B V  4LRXA                282    0.025   3.796   0.791   0.002   0.816   0.182
B N  4LRXA                283    0.104  15.270  -0.319   0.005   0.336   0.660
B N  4LRXA                284    0.280  40.948  -1.454   0.018   0.047   0.935
B L  4LRXA                285    0.181  33.086  -1.390   0.018   0.019   0.964
B G  4LRXA                286    0.257  20.242  -1.998   0.005   0.015   0.979
B A  4LRXA                287    0.179  19.748  -1.878   0.016   0.005   0.979
B T  4LRXA                288    0.061   8.405   0.205   0.109   0.005   0.886
B P  4LRXA                289    0.171  24.208   0.194   0.176   0.004   0.820
B L  4LRXA                290    0.182  33.306  -0.177   0.923   0.002   0.076
B S  4LRXA                291    0.050   5.883   0.056   0.970   0.001   0.030
B E  4LRXA                292    0.031   5.468  -1.163   0.970   0.001   0.030
B L  4LRXA                293    0.031   5.639  -0.192   0.970   0.001   0.030
B Y  4LRXA                294    0.068  14.596  -0.099   0.970   0.001   0.030
B G  4LRXA                295    0.037   2.928  -0.016   0.988   0.000   0.012
B V  4LRXA                296    0.026   3.996   0.307   0.970   0.001   0.030
B Y  4LRXA                297    0.047  10.065   0.726   0.970   0.001   0.030
E N  4LRXA                298    0.284  41.519   0.937   0.970   0.001   0.030
E R  4LRXA                299    0.282  64.555   1.621   0.988   0.000   0.012
B L  4LRXA                300    0.015   2.746   1.414   0.988   0.000   0.012
B T  4LRXA                301    0.141  19.529   1.460   0.970   0.001   0.030
E T  4LRXA                302    0.563  78.116   1.971   0.970   0.001   0.030
B R  4LRXA                303    0.194  44.380   1.939   0.970   0.001   0.030
B C  4LRXA                304    0.027   3.861   0.506   0.970   0.001   0.030
E Q  4LRXA                305    0.602 107.517   1.365   0.858   0.002   0.139
E Q  4LRXA                306    0.803 143.469   1.269   0.694   0.003   0.303
E A  4LRXA                307    0.388  42.769   1.548   0.109   0.005   0.886
E G  4LRXA                308    0.745  58.624   0.449   0.003   0.003   0.994
B L  4LRXA                309    0.051   9.375   0.680   0.005   0.045   0.951
E T  4LRXA                310    0.456  63.233   2.006   0.003   0.718   0.279
B I  4LRXA                311    0.074  13.690   0.694   0.001   0.900   0.099
E E  4LRXA                312    0.252  44.042   0.527   0.001   0.900   0.099
B R  4LRXA                313    0.226  51.846   0.712   0.011   0.918   0.071
B N  4LRXA                314    0.091  13.381   0.200   0.018   0.846   0.136
B L  4LRXA                315    0.139  25.451   0.140   0.023   0.655   0.322
B I  4LRXA                316    0.081  14.929  -0.830   0.023   0.655   0.322
B G  4LRXA                317    0.072   5.659  -0.868   0.069   0.386   0.545
E A  4LRXA                318    0.271  29.853  -0.303   0.125   0.227   0.648
B Y  4LRXA                319    0.073  15.707   0.295   0.148   0.418   0.435
B C  4LRXA                320    0.067   9.351   0.614   0.159   0.516   0.325
B T  4LRXA                321    0.082  11.332  -0.711   0.159   0.516   0.325
B S  4LRXA                322    0.051   6.012  -0.851   0.135   0.317   0.548
B L  4LRXA                323    0.072  13.202  -0.063   0.118   0.150   0.732
E D  4LRXA                324    0.330  47.596  -0.596   0.053   0.043   0.903
B M  4LRXA                325    0.108  21.551  -0.955   0.058   0.017   0.925
E T  4LRXA                326    0.228  31.554  -0.905   0.053   0.043   0.903
B G  4LRXA                327    0.064   5.045  -1.727   0.064   0.216   0.721
B F  4LRXA                328    0.037   7.326   0.052   0.023   0.655   0.322
B S  4LRXA                329    0.046   5.415   0.121   0.018   0.846   0.136
B I  4LRXA                330    0.026   4.884   0.916   0.011   0.918   0.071
B T  4LRXA                331    0.049   6.782  -0.251   0.011   0.918   0.071
B L  4LRXA                332    0.040   7.416  -0.111   0.018   0.846   0.136
B L  4LRXA                333    0.060  11.004   0.360   0.021   0.756   0.223
E K  4LRXA                334    0.335  68.827   0.928   0.021   0.451   0.528
B V  4LRXA                335    0.058   8.853   0.740   0.019   0.141   0.840
E D  4LRXA                336    0.348  50.219   0.956   0.113   0.043   0.844
E D  4LRXA                337    0.706 101.720   0.845   0.923   0.002   0.076
E E  4LRXA                338    0.494  86.302   1.211   0.923   0.002   0.076
B T  4LRXA                339    0.058   7.975   1.041   0.970   0.001   0.030
E L  4LRXA                340    0.225  41.161   1.854   0.970   0.001   0.030
E A  4LRXA                341    0.556  61.260   1.898   0.970   0.001   0.030
B L  4LRXA                342    0.071  12.982   0.915   0.923   0.002   0.076
B W  4LRXA                343    0.058  13.925   0.575   0.858   0.002   0.139
E D  4LRXA                344    0.552  79.500   0.336   0.502   0.002   0.495
B A  4LRXA                345    0.194  21.335   1.370   0.109   0.005   0.886
E P  4LRXA                346    0.356  50.559   0.349   0.115   0.016   0.868
B V  4LRXA                347    0.078  11.973  -0.052   0.115   0.016   0.868
E H  4LRXA                348    0.559 101.591  -0.007   0.113   0.043   0.844
B T  4LRXA                349    0.069   9.556   0.285   0.058   0.017   0.925
E P  4LRXA                350    0.400  56.774  -0.736   0.058   0.017   0.925
E A  4LRXA                351    0.386  42.482  -1.843   0.018   0.019   0.964
B L  4LRXA                352    0.174  31.878   0.446   0.018   0.088   0.893
E N  4LRXA                353    0.452  66.187  -0.274   0.019   0.141   0.840
E W  4LRXA                354    0.321  77.104  -0.099   0.018   0.047   0.935
E G  4LRXA                355    0.502  39.500  -2.499   0.018   0.019   0.964
E K  4LRXA                356    0.869 178.733   0.280   0.003   0.003   0.994

Explain the output.  Go back.
```

---
